# Eco‐oncology: Applying ecological principles to understand and manage cancer

**DOI:** 10.1002/ece3.6590

**Published:** 2020-07-29

**Authors:** Brent A. Reynolds, Monika W. Oli, Madan K. Oli

**Affiliations:** ^1^ Department of Neurosurgery College of Medicine University of Florida Gainesville FL USA; ^2^ Department of Microbiology and Cell Science Institute of Food and Agricultural Sciences University of Florida Gainesville FL USA; ^3^ Department of Wildlife Ecology and Conservation Institute of Food and Agricultural Sciences University of Florida Gainesville FL USA

**Keywords:** cancer, cancer ecology, cell to cell communication, chaos, complex adaptive systems, cytotoxic cancer therapies, eco‐oncology, ecological therapy, ecology, evolution of resistance, metastasis, non‐linear dynamics, oncology

## Abstract

Cancer is a disease of single cells that expresses itself at the population level. The striking similarities between initiation and growth of tumors and dynamics of biological populations, and between metastasis and ecological invasion and community dynamics suggest that oncology can benefit from an ecological perspective to improve our understanding of cancer biology. Tumors can be viewed as complex, adaptive, and evolving systems as they are spatially and temporally heterogeneous, continually interacting with each other and with the microenvironment and evolving to increase the fitness of the cancer cells. We argue that an eco‐evolutionary perspective is essential to understand cancer biology better. Furthermore, we suggest that ecologically informed therapeutic approaches that combine standard of care treatments with strategies aimed at decreasing the evolutionary potential and fitness of neoplastic cells, such as disrupting cell‐to‐cell communication and cooperation, and preventing successful colonization of distant organs by migrating cancer cells, may be effective in managing cancer as a chronic condition.

## Graphical abstract

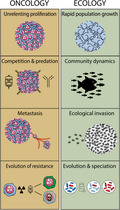

## 1 INTRODUCTION

Cancer is one of the leading causes of human morbidity and mortality worldwide. Collectively, there are about 14 million new cases around the world, and over 8 million cancer‐related deaths per year (McGuire, 2016). Globally, the incidence is not declining and is expected to rise to 22 million new cases per year (a 70% increase) in the next two decades. In the United States, cancer is a significant health problem, with 1 in 4 deaths being due to cancer and male and female Americans having 44% and 38% chance of developing cancer during their lifetimes, respectively. The incidence of cancer has been increasing, while mortality has been decreasing since the mid‐1970s (Siegel et al., 2014). Although progress in increasing life expectancy is being made, the progress pales in comparison to advances made with other major diseases like heart disease and stroke (Hole & Salem, 2016). While heart disease has been the leading cause of death in the USA over the past 50 years, this is expected to change in the coming decades.

The term “cancer” is used to describe a large number of diseases that can affect nearly every part of the body. What they share in common is the uncontrolled growth of cells and tissues as a result of unmitigated tumor cell proliferation. Cancer often arises from a single cell that is transformed in a multi‐step process beginning with a normal cell that develops into a precancerous lesion and eventually evolves into a malignant tumor. In broad terms, causation is an interaction between a person's genetic makeup and external influences such as physical, chemical, and biological carcinogens, with the most influential risk factor being aging. However, the intricacies of a cell developing from a normal cell into a cancerous mass are complex and hold many clues as to how one can intervene in this process. In 2000 and then in 2011, Hanahan and Weinberg (Hanahan & Weinberg, 2000, 2011) proposed several hallmarks of cancer to provide a framework to better understand the diversity of neoplastic diseases and provide a foundation for the understanding of characteristics shared by most tumor populations.

One of the hallmarks, likely the most fundamental property of neoplastic diseases, is relentless and chronic cell proliferation, which contrasts with healthy tissue where cell turnover and replacement of damaged cells are well‐controlled, involving an orchestrated mix of growth‐promoting and inhibiting signals, ensuring the tissue retains its normal architecture, size, and function. Through a sequence of transforming events, cancerous‐like cells lose this system of checks and balances, which is so necessary for tissue maintenance, and instead, exhibit relentless growth that involves two distinct processes. The first is the activation of growth‐promoting signals. This is part of a complex web that involves multiple mechanisms, including autocrine overproduction of growth‐promoting signals, deregulated receptor signaling, and interference in negative feedback that would diminish proliferation signaling. Surprisingly, tumor cells can communicate with the surrounding "normal" tissue which in turn plays a supportive role in promoting tumor cell proliferation (Bhowmick, Neilson, & Moses, 2004; Cheng et al., 2008; Yasuda et al., 2014). The second process involves tumor suppressor genes that sense intracellular and extracellular signals, DNA damage, and nutrient levels, integrate this information, and decide whether or not a cell should progress through the cell cycle. The loss of activity of tumor suppressor genes, often due to genetic mutations or epigenetic silencing, is a common event in many cancers (Kazanets, Shorstova, Hilmi, Marques, & Witcher, 2016; Wang, Wu, Rajesekaran, & Shin, 2018).

A further hallmark is the ability of tumor cells to invade surrounding tissue and metastasize to distant sites. In culture, noncancerous cells exhibit "contact inhibition” where cell‐to‐cell contact can suppress cell proliferation. In vivo, this plays a crucial role in maintaining tissue homeostasis. The loss of contact inhibition, the ability to escape the local environment, and establish a new home in a distant location is a defining characteristic of nearly all advanced cancers. Without this characteristic, tumors stay confined to a particular location and are more amenable to physical removal. The ability of tumor cells to escape their local niche is a well‐orchestrated multi‐step event referred to as the invasion–metastasis cascade (Lloyd, Gatenby, & Brown, 2017; Valastyan & Weinberg, 2011) and begins with invasion of the surrounding environment, intravasation into the surrounding lymph or blood vessels, distant movement from their primary site of growth and then extravasation from blood and lymph vessels back into other tissues. At this point, the migrating cells set up small colonies that attempt to establish themselves as metastatic tumors. Key to this process is the reinitiation of a developmentally regulated program called "epithelial–mesenchymal transition" [EMT], which involves a sequence of events that allow epithelial cells to take on characteristics of mesenchymal cells and gain migratory and invasive properties (Nieto, Huang, Jackson, & Thiery, 2016; Pastushenko & Blanpain, 2019). The local environment (stromal cells and the invasive margins of a tumor) and the immune system are believed to play a role in activating a number of the genes responsible for the EMT program and modifying the surrounding environment making it permissive for invasive growth, respectively. However, escape and distant migration are only half of the story; successful colonization still needs to occur and is not guaranteed. It is well established that patients can have multiple micrometastasis that report on the migration of tumor cells from their primary site but never establish a macroscopic tumor or successful secondary colonies. Adaptation of a tumor cell to a new environment was eloquently described in 1889 by Stephen Paget (Paget, 1889) when he advanced the "seed and soil" theory of metastasis, proposing that tumor cells [the seed] interact with its metastatic site [the soil] and that successful colonization was dependent on both the seed and soil being receptive to new growth. This idea has held up well, and today it is well accepted that the metastatic process selects for cells that undergo several challenging processes (i.e., EMT, invasion, embolization, circulatory survival, extravasation) and that the host tissue needs to be receptive to these cells (Paget, 1889; Robatti, Mangialardi, & Vacca, 2006). This latter point is evident clinically in the observation that certain types of cancer preferentially metastasize to specific organs. Hence, the outcome of metastasis is dependent on multiple interactions among tumor cells, the stromal and the new microenvironment, which is continuously modified as the neoplastic progression advances (Joyce & Pollard, 2009; Wang et al., 2017).

Cell death is a necessary process that helps shape our body during development, plays a crucial role in maintaining tissue architecture, and is a mechanism to eliminate cells that are not functioning correctly or have been damaged due to stress, nutrient deprivation, or viral infection. This type of cell death is referred to as apoptosis or programmed cell death and is a kind of “cell suicide” that cells initiate when normal function has been significantly compromised (Lee et al., 2018). While many of the events that occur in a tumor cell would initiate apoptosis, cancers often evade this fundamental regulatory mechanism (Evan & Vousden, 2001; Gerl & Vaux, 2005). A second type of cell death is necrosis. Unlike the more orderly and reversible apoptosis, necrosis tends to be a one‐way event, has been traditionally thought to be caused by external influences such as trauma, toxins or external cell signaling, and often invokes a proinflammatory response that can recruit tumor‐promoting inflammatory cells, stimulate tumor cell proliferation, foster tumor cell invasion and encourage, one of the hallmarks of cancer, sustained angiogenesis (Lee et al., 2018). Central to the role that necrosis can play in boosting cancer growth is its participation in the cascade of events related to inflammation which occurs as a result of attracting tumor stimulating inflammatory cells and releasing cytokines that can induce proliferation of neighboring tumor cells (Labi & Erlacher, 2015; Lee et al., 2018). Hence, necrosis of cancer cells, as a result of endogenous mechanisms or treatments such as chemotherapy and radiation, can cause a significant amount of tumor cell death, but it can also be tumor‐promoting and can ultimately do more harm than good.

A key hallmark of cancer is the immortal nature of cancer cells. In the 1960s, Leonard Hayflick demonstrated that normal human fetal cells would divide between 40 and 60 times in culture, after which the cells entered a nonproliferative senescence phase or a crisis state leading to cell death (Bodnar et al., 1998). This phenomenon, referred to as the “Hayflick limit” (Hayflick & Moorhead, 1961), is due to the shortening of telomeres that protect the ends of chromosomes. Each cell division results in the erosion of telomeres leading to senescence or a crisis state. Telomerase is an enzyme that adds new nucleotides to the ends of telomeres, extending the cells’ ability to proliferate past the Hayflick limit. Telomerase activity is nearly absent in healthy cells but is highly expressed in many cancer cells (but see Haussmann et al., 2007). Hence, the ability of cancer cells to upregulate telomerase activity and its ability to counter telomere erosion provides cancer cells with a limitless proliferative ability, thereby making them immortal (Armstrong & Tomita, 2017; Francica, Aebersold, & Medová, 2017).

## 2 EVOLUTIONARY ECOLOGY OF CANCER

Darwinian evolution can be viewed as a change over time in heritable characteristics of biological populations that occur at a species, organism, cellular, or even a molecular level. In multicellular organisms, cells cooperate and collectively promote survival and reproductive success of the whole organism to promote the replication of shared genetic material. Once in a while, however, somatic mutations allow cells to increase their fitness at the expense of the well‐being and fitness of other cells or populations, and in some circumstances, even the whole organism. Adaptation, speciation, anagenesis, and extinction are responsible for the diversity of life on our planet and have a direct impact on all areas of biology. In cancer, the accumulation of several mutations and epigenetic alterations (known as the Knudson hypothesis) (Knudson, Di Ferrante, & Curtis, 1971; Nordling, 1953) generate genetic heterogeneity, with subclones exhibiting unique abilities to survive and proliferate. Thus, the necessary and sufficient conditions for natural selection – variation in traits, heritability and fitness difference – are present in neoplasm (Greaves & Maley, 2012; Lloyd et al., 2017; Nowell, 1976) and set the stage for neoplastic cells to progressively acquire the hallmark capabilities described above (Hanahan & Weinberg, 2000, 2011). Acquisition and expression of these capabilities are facilitated by genomic instability that permits multi‐stage mutations and epigenetic alterations, thus creating genetic diversity and somatic selection for phenotypes that are capable of expressing cancer's hallmark characteristics and progressively achieving a neoplastic state (Maley et al., 2017). The ability of tumor cells to adapt to changing circumstances is remarkable. For instance, as a result of the tumor cells’ rapid proliferation, they quickly outgrow their blood supply, create a hypoxic environment and require large quantities of macromolecules to be incorporated into their biomass for new cell generation. In response, cancer cells can switch energy metabolism from mitochondrial oxidative phosphorylation to aerobic glycolysis, a process referred to as the Warburg Effect (Warburg, 1956a, 1956b). While this is a less efficient method for generating energy it is necessary for biomass incorporation. During this process, tumor cells produce lactic acid, which alters the microenvironment in a manner that makes it more favorable for tumor cell growth and expansion (Ibrahim‐Hashim, Gillies, Brown, & Gatenby, 2017). This alteration of the tumor microenvironment (“niche construction”) via altered energy metabolism is thought to be an essential process leading to tumor cell progression (Warburg, 1956a, 1956b). Thus, cancer is driven primarily by somatic (or clonal) evolution of cell lineages which have escaped mechanisms that control cellular replication and acquired capabilities that allows them to increase their fitness (Crespi & Summers, 2005; Ducasse et al., 2015; Gillies, Verduzco, & Gatenby, 2012; Merlo, Pepper, Reid, & Maley, 2006; Nowell, 1976). Genetically and phenotypically cells within a tumor are changing over time in response to an assortment of influences such as cancer treatments, the immune system, access to nutrients and alteration of the TME as a result of cell proliferation and cell death. These are central to the core of cancer evolution, and a better understanding and therapeutic targeting of each of these components can help the design of more effective treatments (Maley et al., 2017; Nesse, 2017).

Adaptation of a species to a changing environment is key to its long‐term survival and evolution. It is well known that resistance to antibiotics in pathogens (and to insecticides in insect pests) evolves employing natural selection (Baquero & Blázquez, 1997; Davies & Davies, 2010). Antibiotics (or pesticides) act as agents of selection by killing individuals that are susceptible to antibiotics (or pesticides), thereby conferring a competitive advantage to individuals that are resistant to antibiotics. Repeated and often indiscriminate application of antibiotics selects for multidrug‐resistant pathogens, which has become a significant challenge for public health. The evolution of resistance to cytotoxic therapies occurs similarly but more rapidly. Standard of care cancer treatments such as chemotherapy and radiation can be effective in killing cancer cells; however, these treatments act as agents of selection, selecting for treatment‐resistant phenotypes. Over time, this phenotype dominates the tumor population. The evolution of resistance to standard of care treatments is a notable roadblock to curing cancer (Foo & Michor, 2014; Gonzalez‐Angulo, Morales‐Vasquez, & Hortobagyi, 2007), yet there exists no plausible way of circumventing this evolutionary process (Gatenby & Brown, 2018). Whereas genomic instability leading to cumulative mutations (aided by epigenetic alterations) continuously creates genetic diversity and heterogeneity in cancer cells, it is the tumor microenvironment and cytotoxic therapies that act as selection forces favoring cellular traits that confer the highest fitness in that particular environment (Daoust, Fahrig, Martin, & Thomas, 2013). Thus, the evolution (clonal, trait, or macro‐evolution heading to speciation) of drug‐resistant phenotypes occurs in an ecological context, with the tumor microenvironment and cytotoxic therapies (i.e., agents of selection) playing profound roles (Aktipis & Nesse, 2013).

An alternative view recently proposed by Sottoriva et al. (2015) postulates that “… tumors grow predominantly as a single [clonal] expansion producing numerous intermixed subclones that are not subject to stringent selection.” This idea, dubbed “Big Bang model”, assigns a relatively minor role to the tumor microenvironment and natural selection, but rather suggests that tumors are “born to be bad”, and its malignant potential is determined during early stages of tumorigenesis (Sottoriva et al., 2015; Robertson‐Tessi & Anderson, 2015). While the Big Bang theory offers a potent alternative to the traditional model of tumorigenesis that emphasizes clonal selection (Nowell, 1976), it suffers from obvious shortcomings. For example, this idea cannot explain the evolution of resistance to cytotoxic therapies, and is inconsistent with the evidence that tumor cells affect, and are affected by, the tumor microenvironment, and with the evidence that the cancer cell‐microenvironment interactions play an important role in tumorigenesis and metastasis (e.g., Aktipis & Nesse, 2013; Joyce & Pollard, 2009; Wang et al., 2017). Finally, in general, the notion that tumors are “born to be bad” is not compatible with current cell biological, oncological, or eco‐evolutionary thinking.

## 3 WHY SHOULD ONCOLOGISTS THINK LIKE ECOLOGISTS?

The striking similarities between ecological populations and communities, and tumors (Table 1) prompts one to evaluate what reciprocal lessons either biological system can teach each other. Pondering cancer from an ecological perspective may improve our understanding of the structure and function of tumors, and help develop or refine integrative therapeutic approaches for several reasons.

**TABLE 1:** Analogies between ecological concepts and cancer biology^a^

Concept	Ecology	Cancer cell biology
**General**		
Population	Collection of individuals of the same species coexisting at the same time and place	Collection of cancerous cells of the same phenotype coexisting at the same time and within a tumor
Community	Collection of interactive populations of different species coexisting at the same time and place	Collection of interactive populations of healthy cells, and cancerous cells of different phenotypes coexisting at the same time within a tumor
Ecological invasion	Invasion of a new habitat by non‐native species. Successful ecological invasion is a multi‐stage process, involving: (1) departure from the native habitat, (2) transportation/dispersal via unsuitable matrix, (3) evasion of natural enemies during transportation/dispersal, (4) colonization of and establishment in the destination habitat, and (5) population growth and range expansion	Metastasis, which is the spread of cancer from the primary tumor to new organs within a host. Successful metastasis is a multi‐stage process, involving: (1) shedding of the cancer cells from the primary tumor, (2) invasion of bloodstream (intravasation) and transportation of circulating cancer cells; (3) evasion of immune system, (4) withdrawal from the bloodstream (extravasation), (4) successful colonization of the new organ, and (5) angiogenesis and tumor growth in the new organ or location
Ecosystem	A community of living organisms along with nonliving environment interacting with each other via exchange of energy and matter	A community of healthy and cancerous cells along with nonliving tumor microenvironment (extracellular matrix, and soluble factors such as glucose and other nutrients, signaling factors, growth factors) interacting with each other via exchange of energy and matter
**Population ecology:**		
Population size	Number of individuals in a population at a given time	Tumor size or volume (or number of tumor cells) within an organ at a given time
Birth rate	Number of births individual^−1^ time^−1^	Number of cell divisions parental cell^−1^ time^−1^
Death rate	Number of deaths individual^−1^ time^−1^	Number of cell deaths parental cell^−1^ time^−1^
Natal dispersal	Number of dispersers individual^−1^ time^−1^	Number of migrating or circulating cancerous cells parental cell^−1^ time^−1^
Population growth rate	Growth rate of a population; depends on the balance between gain (from births and immigration) and loss (from death and dispersal) rates	Growth rate of a tumor; depends on the balance between gain (from cell division) and loss rates (cell death and dispersal or emigration)
Intraspecific competition	Competition among individuals of the same species	Competition among cells of different cancerous phenotypes within a tumor
Interspecific competition	Competition among individuals of different species	Competition among cancerous and normal (healthy) cells within a tumor
Density‐dependence	Dependence of population growth rate on present or past population density due to space and resource limitations	Dependence of tumor growth rate on available space and resources within an organ due to space and resource limitations
Carrying capacity	The maximum number of individuals an environment can support without destroying the environment	The maximum tumor volume an organ can support without causing serious damage to the organ itself or killing the host individual
Metapopulation	A population of populations connected through exchange of individuals	A collection of tumors of the same kind with possible exchange of cancer cells among tumors
Source population	A population with positive growth that can persist without immigration; emigrants can disperse to other subpopulations or colonize empty habitat patches	Primary tumors (a tumor growing within an organ where tumor progression began and proceeded to develop into a tumor); migrants leaving the primary tumors can colonize (or metastasize) in other organs
Habitat patch	A patch of suitable habitat where individuals can survive and reproduce	Organs or tissues where cancer cells can proliferate and form tumors; the “soil” of the “seed and soil” theory of metastasis
Matrix	Hostile landscape that is unsuitable for individuals for survival or reproduction but one that can be used by animals for dispersal or migration	Parts of the host individual's body or organs where cancer cells cannot proliferate but through which they can travel (e.g., bloodstream)
Propagules	Dispersing individuals or seeds that can potentially colonize vacant habitats	Circulating cancer cells that can metastasize in host organs
**Community ecology**		
Species richness	Number of species in an ecological community	Number of cancer cell genotypes and phenotypes within a tumor (intratumoral heterogeneity)
Interspecific competition	Competition among individuals of different species for space and resources	Competition between normal and cancerous cells within a tumor microenvironment. Within a tumor, competition between cells with aerobic and anaerobic metabolism; and between treatment‐resistant and nonresistant cancer cells
Predation	One species consuming another	Destruction of cancer cells by immune system or cytotoxic therapies
Mutualism	Mutually beneficial interactions among individuals of different species	Heterogeneous collections of cells within a tumor cooperating with each other to evade immune response and promote tumor growth
Propagules	Dispersing individuals that are capable of long‐distance dispersal and thus can potentially colonize new habitats	Circulating cancer cells that can potentially colonize new organs (metastasis)
**Evolutionary Ecology**		
Phenotypic variation	Variation among individuals due to germline mutation, recombination, and phenotypic plasticity. Each population is composed of genetically divergent individuals with differential ability to survive and reproduce	Somatic mutation, phenotypic plasticity, and epigenetic alteration leading to intratumoral heterogeneity. Each tumor is composed of different cancerous cell genotypes and phenotypes with differential abilities to survive and proliferate
Fitness	Rate at which genotypes (or phenotypes) are represented in future generations. Determined by survival and reproductive success	Rate at which cancer cell genotypes (or phenotypes) are represented in future generations of cancerous cells at the primary or metastatic tumor. Determined by survival and rate of proliferation of cancer cell genotypes (or phenotypes)
Inheritance	Genes passed on to offspring unaltered, except those arising from mutation and recombination	Genes passed unaltered from parent cancer cells to daughter cells, except alterations due to somatic mutation or epigenetic alteration
Evolution of resistance	Natural selection favoring genotypes that are resistant to antibiotics or pesticides. Some individuals within a population are resistant to antibiotics/pesticides and others are not (variation in trait); offspring of resistant individuals tend to be resistant (inheritance); and resistant individuals survive better and thus have higher fitness when subjected to antibiotics or pesticides (fitness difference). Thus, all necessary and sufficient conditions for trait evolution exist, and antibiotics or pesticides act as the agent of selection	Natural selection favoring neoplastic genotypes/phenotypes that are resistant to cytotoxic therapies. Some cancer cells are resistant to cytotoxic therapies and others are not (variation in trait); daughter cells of resistant parental cells tend to be resistant (inheritance); and resistant cells survive better and thus have higher fitness when subjected to cytotoxic drugs (fitness difference). Thus, all necessary and sufficient conditions for trait evolution exist, and cytotoxic therapies act as agents of selection
Life history traits	Traits of organisms that directly influence individual fitness (e.g. survival and reproductive rates, age of first or last reproduction)	Traits of cells that directly influence cellular fitness (e.g., cellular survival and proliferative rates, cellular age of first or last cell division)
Life history trade‐offs	Trade‐off among fitness traits such that increase in fitness due to a beneficial change in one trait is counteracted by a decrease in fitness due to a detrimental change in another trait	The existence of therapy‐resistant “slow‐cycling” cancer stem cells represent a population of tumor cells that trade‐off proliferation for increased survival when subjected to cytotoxic therapies (i.e. chemotherapy or radiation)

^a^ Sources: Aktipis (2016); Aktipis et al. (2013); Boddy, Huang, & Aktipis (2018); Bowler & Benton (2005); Crespi & Summers (2005); Deleyrolle et al. (2011); Gatenby (1996); Gatenby & Brown (2018); Greaves (2013); Greaves & Maley (2012); Kareva (2011b); Korolev, Xavier, & Gore (2014); Krebs (2001); Lockwood et al. (2013); Maley et al. (2017); Merlo et al. (2006); Moore, Wells, Van Vuren, & Oli (2016); Nesse (2017); Nesse, Stearns, & Omenn (2006); Roff (2010); Oli (2004); Oli & Coulson (2016); Stearns (1989, 1992); Thomas et al. (2013); Ujvari, Roche, & Thomas (2017a), Ujvari, Roche, & Thomas (2017b); Valastyan & Weinberg (2011).

First, tumors are inherently complex and evolving ecological systems, with multifaceted interactions among biotic (tumor cell phenotypes, healthy cells, stromal cells, killer lymphocytes, vasculature) and abiotic (extracellular matrix, and soluble factors such as glucose and other nutrients, signaling factors, growth factors) components of the microenvironment. Cancer cells interact with both biotic components of the tumor microenvironment through interactions such as “predation” by the immune system or cancer therapies, and competition for resources between cancer and healthy cells, and among cancer cell phenotypes. Many animals live or travel in groups as the risk of individual predation is reduced as group size increases (Foster & Treherne, 1981; Mooring & Hart, 1992); similarly migration of cancer cells in groups from a primary tumor allows them to more effectively evade the immune system thereby increasing the likelihood of metastasis (Deisboeck & Couzin, 2009). There exists evidence that cancer cells cooperate, using mechanisms such as diffusible factors to promote neoplastic progression, and they can even recruit noncancerous stromal cells to support tumor growth (Axelrod, Axelrod, & Pienta, 2006; Joyce & Pollard, 2009). Such biotic interactions are analogous to mutualistic and commensalistic interactions in ecological communities (Mittelbach, 2012). All living components within tumors also interact with the abiotic tumor microenvironment, with a constant flow of energy and matter between “biotic communities,” and the abiotic tumor microenvironment (Aktipis & Nesse, 2013; Basanta & Anderson, 2013; Chen & Pienta, 2011; Mittelbach, 2012). Indeed, a tumor can be thought of as a complex ecosystem embedded within organs of multicellular organisms; understanding the structure and function of such a system necessitates a thorough understanding of components of the system and interactions among them (Chen & Pienta, 2011; Greaves & Maley, 2012).

Secondly, tumor growth and metastasis are essentially population ecological problems where the focus is to understand factors and processes that drive the changes in tumor size or volume over time and space. During early stages of carcinogenesis, tumor cell populations grow rapidly according to the exponential growth model: $\frac{\mathrm{dN}}{\mathrm{dt}}=rN,$ where *r* is the tumor growth rate, *N* is the number of tumor cells or volume occupied by a tumor, and *dN*/*dt* is the rate of change in tumor size or volume (or number of tumor cells). As the tumor expands, space within the organ, as well as the supply of blood and nutrients, become limiting. Consequently, the tumor growth rate slows, and ultimately ceases, due to the lack of space and/or resources. This phenomenon is succinctly described by the logistic population growth model: $\frac{\mathrm{dN}}{\mathrm{dt}}=rN\left(1‐\frac{N}{K}\right),$ where *K* is the carrying capacity of the tumor microenvironment. When the tumor size or volume (or the number of cancer cells) reaches *K*, tumor growth ceases. The population growth rate, as well as the carrying capacity, can vary spatially, especially in tumors that originate in confined anatomical structures (e.g., breast cancer; Gerlee & Anderson, 2015).

Within a tumor, subpopulations, or regions of spatial heterogeneity may exist exhibiting different survival and proliferative abilities, a situation akin to demographically or spatially structured population dynamics in ecology (Dagogo‐Jack & Shaw, 2017). In ecological populations, individuals of different age or life‐history stages, or those inhabiting different habitats may exhibit a different propensity to survive or reproduce, causing age‐, stage‐, or location‐specific differences in demographic rates. Dynamics of populations composed of heterogeneous individuals are modeled using spatially or demographically structured matrix (exponential or density‐dependent populations) population models (Caswell, 2001). It is now widely recognized that while cells within tumors are heterogeneous, so too is the tumor microenvironment (Runa et al., 2017; Wang et al., 2017). Hence, it is logical to presume that tumor cell proliferation can differ widely even within a tumor depending on the cell genotype or phenotype and local microenvironment. Spatial heterogeneity in birth and death rates are facts of life in ecology and are typically studied within the framework of demographically‐ or spatially‐structured population dynamics (e.g., Caswell, 2001; Hanski, 1999). Likewise, the proliferation rate of cancer cells within a single tumor can vary considerably, dependent on the cell genotype or phenotype and the local tumor microenvironment or niche where the cells are located. In addition, primary and metastatic tumors may interact via circulating cancer cells, a situation identical to metapopulation systems in ecology (González‐García, Solé, & Costa, 2002).

Thirdly, disseminated cancers can be thought of as biological invasions as both share many common features (Gatenby, Brown, & Vincent, 2009; Gatenby, Silva, Gillies, & Frieden, 2009; Lloyd et al., 2017). Biological invasion occurs when a species colonizes a novel but suitable habitat away from its native range (Lockwood, Hoopes, & Marchetti, 2013; Shigesada & Kawasaki, 1997). If the new environment is devoid of natural enemies or is otherwise favorable, and the species possesses characteristics for it to become a successful invader, the stage is set for ecological invasion—it can spread quickly, taking over vast areas and causing extensive ecological damage including decimation of native prey species, competitive exclusion of ecologically similar native species and alteration of the microenvironment. Ecological theory proposes the success of a biological invasion depends both on characteristics of the invaders that make them successful and the invasibility of the environment. Many successful invaders (plants, animals, or microorganisms) share characteristics that allow for rapid colonization and range expansion. These characteristics include (but are not limited to): fast growth and maturation, early reproduction (sexual and/or asexual), rapid population growth (owing to rapid proliferation, vegetative propagation and/or a large number of offspring per reproductive attempt), long‐distance dispersal capabilities, resistance to mechanical or chemical control measures, and adaptability and capability to alter the environment to favor itself at the expense of potential competitors (Lodge, 1993; Shea & Chesson, 2002; Shigesada & Kawasaki, 1997). This situation is strikingly similar to cancer metastasis with circulating cancer cells serving as propagules that spread from their primary site and ultimately colonize a new site (Chen & Pienta, 2011); indeed, this idea is embedded in the “seed and soil” theory of metastasis (Paget, 1889). Disseminated cancers can be thought of as biological invasions because these two processes share many common features (Table 1). As cancer cells are dislodged from a tumor and enter the bloodstream, some of the circulating cancer cells evade the immune system, establish themselves in a new environment, proliferate, and form secondary tumors. After tumor cells have begun invading a new site, they attract vasculature (angiogenesis) to ensure a supply of oxygen and nutrients, and when faced with a hypoxic environment, they switch energy metabolism to glycolysis. In this process, they alter tumor microenvironment by producing lactic acid and other metabolites that can assist with their survival and proliferation. This strategy, commonly called “niche construction,” is employed by many types of cancers (Kareva, 2011b; Polyak, Haviv, & Campbell, 2009), as well as many invasive species (Gordon, 1998; Kareva, 2011a, 2011b). Unstable and disturbed ecosystems with empty niches are more likely to be invaded by exotic invaders; likewise, cancer has been described as an emergent property of disturbed, resource‐rich environments (Ducasse et al., 2015).

Adler and Gordon (2019) recently pointed out important differences between cancer and ecological invasions. Such differences are to be expected in complex natural systems; no two cancers metastasize in the same manner, and every ecological invasion is unique in some way. Despite these differences, and uniqueness of each system, fundamental principles driving cancer metastases are essentially the same as those underlying successful ecological invasions (Table 1). For example, the metastatic cascade involves shedding of cancer cells from the primary tumor, invasion into the bloodstream (intravasation), transportation of circulating cancer cells and evasion of the immune system, withdrawal from the bloodstream (extravasation), and successful colonization of the new organ, followed by angiogenesis and tumor growth (Lloyd et al., 2017; Paterlini‐Brechot & Benali, 2007). The equivalent processes in ecological invasion involves departure from the native habitat, transportation/movement via an unsuitable matrix, evasion of natural enemies during transportation/dispersal, colonization of and establishment in the destination habitat, followed by population growth and range expansion (Table 1; Blackburn et al., 2011; Lockwood et al., 2013). Most circulating cancer cells do not metastasize; likewise, most ecological invasions fail. Consistent with the seed and soil theory of metastasis (Paget, 1889) the best predictors of the success of ecological invasions are those variables that reflect the interaction between the invading species (seed) and the characteristics of the new habitat (soil) (Romanuk et al., 2009).

Finally, tumors can be thought of as evolving, complex adaptive ecological systems (Miller & Page, 2007; Schwab & Pienta, 1996). A tumor the size of a pea is composed of millions of cells each one acting as an agent with only two purposes: survival and proliferation. There is no evidence that the actions of individual cancer cells are intrinsically motivated to form a tumor, to harm the environment or the host it resides within. Instead, they simply focus on survival and proliferation. Hence, cancer as a disease is an emergent property founded on the interactions of cells or agents with each other and with their microenvironment. Fundamentally, cancer is a disease of single cells that expresses itself at a population level. The sheer number of cells within a solid tissue tumor at the time of detection make it difficult to grasp both conceptionally and practically the contribution of individual cells. Due to this complexity and limitations in seeing the myriad of interactions occurring within such a large population, tumor biology is often studied at the tumor level. This complexity is further confounded by heterogeneity within a tumor (and between tumors of the same classification), making it more difficult, yet seemingly essential and necessary to define, classify and design interventions that reflect intratumoral heterogeneity instead of treating a tumor as a collection of homogenous cancerous cells (Marusyk & Polyak, 2010). A brief description of the salient features of complex adaptive systems (CAS) and how these are exhibited in cancer populations follows (see also, Brownlee, 2007; Miller & Page, 2007; Savit, Riolo, & Riolo, 2013).

### 3.1 Decentralized or distributed control

The analogy that cells within a tumor can be viewed as individual agents similar to ants that make up an ant colony is engaging. In the same sense, the absence of top–down management or the presence of a leader or a master plan, characteristic of a CAS, would also apply to a tumor population. Hence, we would argue that the CAS characteristic of decentralized control is demonstrated by solid tissue tumors.

### 3.2 Emergent properties

Unlike systems composed of independent agents, individual agents in CAS communicate with one another, alter their strategies based on the actions of the other agents, or in response to perturbations to the environment. It is through this process that they learn and evolve and how new system‐level properties, which could not be predicted from the actions of individual agents, emerge. For our purposes, the formation of primary or metastatic tumors, the clinical expression of the disease, and increased robustness and/or resistance to treatment can all be viewed as emergent properties (e. g., Ducasse et al., 2015; Hitomi et al., 2015). While resistant phenotypes and tumor robustness can develop as an emergent property, this does not preclude that each tumor cell operates independently and resistance is the result of a nonresponsive subpopulation that appears as a consequence of tumor heterogeneity. It is essential to note this is very different from the emergent property of a CAS, which occurs because of communication, feedback, and resulting adaptation under selection pressure. Central then to emergent properties of complex adaptive systems is communication among agents (see the Section 3.4 below). While specific details are yet to be established, cancer as a disease can be considered an emergent property due to the interaction among immune system, heterogeneous cancer cell phenotypes, and the biotic and abiotic components of the tumor microenvironment.

### 3.3 Simple rules

Tumor cells have a limited repertoire of behaviors that are elicited by a more complex, but still limited, set of inputs. In this regard, the response of a tumor cell to its environment is simple (although the molecular details of this response are more complex) the CAS approach to tumor management is to shift the reaction of subpopulations of tumor cells to modify the behavior of the entire population.

### 3.4 Connectivity and communication

A vital element of a CAS is how individual agents communicate and interact with each other. This is central to the issue of emergence, coevolution, and the ability of a CAS to adapt to changing circumstances. Without communication and feedback, these processes would not occur. Hence, demonstrating and understanding how this occurs may provide new targets for therapeutic intervention. This is somewhat of a departure from approaches aimed at directly targeting the tumor cells with cytotoxic therapies (radiation, chemotherapy), targeted (receptor or pathway inhibitors) or immunological approaches. A potential mode of communication within solid tissue tumors is intercellular channels that connect the interior of adjacent cells, referred to as gap junctions. Connexin 43 (Cx43) is the main gap junction protein in the brain and is responsible for the extensive coupling of astrocytes (single astrocyte can have 30K gap junction channels). Glioblastoma (GBM) cells have been shown to express Cx43, form homo‐cellular interactions with GBM cells, hetero‐cellular interactions with astrocytes, and demonstrate a positive correlation with Cx43 expression; glioma invasiveness and chemical or peptide blocking of gap junctions (GJ) inhibits migration and sensitizes GBM cells to ligand‐induced apoptosis. Communication with Ca++ signaling occurs via gap junctions in glioma cells and activation of ATP‐sensitive potassium channels can upregulate Cx43 expression and increase gap junction communication, while blockage inhibits proliferation (Hitomi et al., 2015; Princen et al., 2001). Thus, we predict that disruption of cell‐to‐cell communication should reduce tumor robustness, making it more vulnerable to cytotoxic therapies.

### 3.5 Nonlinear dynamics and chaotic behavior

An additional hallmark of complex adaptive systems is nonlinear dynamics and sensitive dependence on initial conditions or response to inputs (more formally, chaos). While linear relationships are often seen in single‐agent studies under highly controlled conditions, nonlinear pharmacodynamics are observed in combination approaches and patient treatments (e.g., Cai et al., 2015; Janecka, 2007; Schwab & Pienta, 1996). This is further supported by the unexpected responses (or lack of response) that are seen in patients. For instance, patients with the same cancer diagnosis often exhibit radically different responses to the same treatment protocol. Even in laboratory experiments, we find statistically different growth rates of human tumor cells that are expanded clonally and then implanted into inbred immuno‐compromised hosts. Hence, the disconnect between therapeutic outcomes, based on well‐executed, experimentally derived expectations, suggests the growth of tumor cells and the response of cancer cells to treatment may be described as nonlinear and chaotic where initial conditions (genetics and physiological state of the patients, the degree of intratumoral heterogeneity) determine the therapeutic outcome (Lowengrub et al., 2010; Orel, Korovin, Molnár, & Orel, 2019).

### 3.6 Coevolution

The interplay between tumor cells and their niche, the tumor microenvironment, is well established (Catalano et al., 2013; Ingber, 2008; Junttila & de Sauvage, 2013; Klein‐Goldberg, Maman, & Witz, 2014; Merlo et al., 2006). Alterations in the microenvironment have been shown to alter brain tumor stem cells, to release molecules that alter the niche in a manner to better support their survival, proliferation and to be protective from radiation and chemotherapy. Different types of cancer cells, healthy cells, and stromal cells interact with each other and alter their actions in response to the actions of normal or stromal cells. As described previously, cancer cells are not only affected by changes in the tumor microenvironment but also actively alter the microenvironment in a way that favors them. In the same vein, the evolution of resistance to cytotoxic therapies, and acquisition and expression of hallmarks of cancer occur as responses to actions of other agents (e.g., immune response, stromal cells) or alteration in the microenvironment (e.g., hypoxia, cytotoxic agents). Collectively, the tumor as a whole can be viewed as a coevolving and coadapting entity. The interplay of communication among a heterogeneous tumor population and its immediate environment is reminiscent of relationships that exist in many biological communities and ecosystems (Levin, 1998).

Thus, all of the salient features of complex adaptive systems are present in tumors, and this can have significant consequences for understanding and managing cancer (Cho, Kim, Kwon, & Kim, 2014). Solid tissue tumors, and cancer as a disease, are emergent properties of interactions among various types of cancer cells, neighboring healthy cells, stromal cells, and the spatially and a temporally heterogeneous tumor microenvironment (i.e., in terms of pH, oxygen and reactive oxygen species concentrations; e.g., Catalano et al., 2013; Junttila & de Sauvage, 2013).

The reductionist approach to understanding natural order in our world has dominated the scientific approach for the past several centuries. Since the time of Descartes, the division of a problem or natural system into as many parts as possible, intending to understand each simple element in detail and then reassembling the pieces step‐by‐step to understand the more complex whole, has defined our scientific method. While scientific reductionism has increased knowledge of many basic principles that define the natural world, it has been conspicuously mute in explaining complex biological systems. Countering the reductionist dogma is the idea that “the whole is greater than the sum of its parts,” an assertion that is central to understanding complex adaptive systems (Miller & Page, 2007; Schwab & Pienta, 1996). An explanation for why reductionism has poorly explained complicated or complex systems is related to the changing behavior and emergent properties of a system composed of many interacting components. In this case, while the cooperating components compose the whole, behavior at the macroscopic level cannot be comprehended by understanding in great detail the workings of each agent; rather, it necessitates an additional understanding of the interactions among the agents, of properties that emerge from these interactions and of how, as a collective unit, the individual agents respond to internal and external influences. In essence, complex adaptive systems are constantly changing and evolving (Deisboeck & Couzin, 2009), presenting somewhat of a moving target when it comes to understanding what makes them tick or how to manipulate them effectively. The evolving nature of such systems results in emergent behavior and is ubiquitously observed in nearly all systems where a large number of elements interact to compose a complex system. Examples of this include the human brain, insect colonies, starling murmurations, stock market investors, and the internet. Just like it is not possible to understand human consciousness by studying individual neurons, similarly, cancer is a disease that may not be amenable to using a reductionist approach. The paradox of studying phenomena at a microscopic level when many of the drivers are operating at a much larger scale may partially explain the general lack of therapeutic improvement made for the majority of cancers.

## 4 THE EDGE OF CHAOS

Building upon work of the famous physicist John von Neumann (von Neumann, 1966) who stated "that there exists a critical [state] below which the process of synthesis is degenerative, but above which the phenomenon of synthesis, if properly arranged, can become explosive," Langton (1990) defined upper and lower limits of complexity where not enough complexity, or too much complexity, produced a degenerative state. Based on studies of cellular automata and spontaneous emerging computation, Langton (1990) found these two states to be close together in the vicinity of a phase transition that he called the edge of chaos. From a biological perspective, this state represents a region of fluidity where apparent chaos creates a highly flexible or adaptable system. A static degenerative state equalizes this chaotic state. The balance between stability and instability, where adequate order is present to maintain the state of the organism, but ample disorder is present to allow sufficient random variations and create a highly adaptable system, defines cancer. Taking the position that cancer lives on the edge of chaos offers two opposing avenues to control tumor growth. First, one can increase instability, via inducing genomic mutations, pushing cells into a degenerative state where they cannot maintain essential function or structure. Second, stability can be promoted via differentiating tumor cells. While the former has yet to be adequately tested, the latter has proven successful in treating blood cancer (de Thé, 2017) and is being tested in several other cancer types (Piccirillo et al., 2006; de Thé, 2017).

## 5 THE CASE FOR ECOLOGICAL CANCER THERAPY

Since the National Cancer Act of 1971, substantial progress has been made in understanding and treating specific cancers, with advances in surgical procedures, and approval of >120 anticancer drugs. Nevertheless, the survival of cancer patients, notably those diagnosed at advanced stages or with metastatic disease, has only improved marginally, despite the introduction of more potent therapies that are effective at killing cancer cells (Weir et al., 200). There are at least two primary explanations to help understand this dichotomy between a plethora of potent cancer drugs and the marginal improvements in cancer outcomes. First, the majority of all cancer therapies are toxic, and aggressive treatment regimens aimed at killing the greatest number of tumor cells also damages and kills healthy cells. This unintended but expected side effect is tempered by dose reduction and treatment suspension (i.e., "drug holiday"), which lowers treatment efficacy. The oncologist seeks a balance between providing the most effective treatment regimen while reducing side effects and maintaining a patient's health and quality of life because cytotoxic drugs can potentially incapacitate or kill the cancer patients before tumors can be annihilated. Thus, physicians either stop treatment or alter the treatment regimen to minimize the side effects. Consequently, while there are several effective cancer‐killing drugs, these agents also kill the patient at doses effective to accomplish their primary intended purpose. These two opposing outcomes become a balancing act in treatment management and one in which the tumor wins for nearly all advanced cancers.

Secondly, it is now well accepted that tumor cell heterogeneity created by genomic instability and epigenetic alterations underlies cancer initiation and tumorigenesis (Merlo et al., 2006; Michor et al., 2005). A tumor starts from a single neoplastic cell and develops into a complex interconnected mass containing billions of cells, with Darwinian evolution playing an essential role during the oncogenesis process (Gillies et al., 2012). Somatic mutations and epigenetic alterations generate intratumoral heterogeneity, and cell phenotypes that are best able to survive and proliferate will be favored by natural selection. Cytotoxic therapies kill therapy‐susceptible cancer cells and thus act as agents of selection favoring therapy‐resistant cancer cell phenotypes. Repeated exposure to these therapies inevitably leads to the evolution of therapy‐resistant cell genotypes, which ultimately dominate the tumor. Therapies become ineffective at that point, likely due to clonal expansion of the resistant population, and then disease relapse (Gatenby & Brown, 2018; Huff, Matsui, Smith, & Jones, 2006; Kareva, Waxman, & Lakka Klement, 2015; Merlo et al., 2006).

The recognition that cancer is a complex, evolving ecological system has led to Darwinian approaches to understanding and treating this disease (Crespi & Summers, 2005, 2006; Greaves, 2007, 2013; Merlo et al., 2006). This manner of thinking has inspired physicians and scientists to consider alternatives to the standard of care treatment regimens based on the maximum dosage of chemotherapy that a patient can tolerate [referred to as maximum tolerated dose (MTD] (Kareva, Morin, & Castillo‐Chavez, 2015; Kareva, Waxman, et al., 2015). For example, *metronomic therapy* is characterized by the administration of cytotoxic drugs and therapies at lower but more frequent doses (Fidler et al., 2000; Hanahan & Weinberg, 2000, 2011; Kareva, Morin, et al., 2015; Kareva, Waxman, et al., 2015; Scharovsky, Mainetti, & Rozados, 2009). This approach focuses on minimizing the toxic effect on patients, reducing the selection pressure for the therapy‐resistant cancer cell phenotypes, and can modify the tumor niche to reduce angiogenesis, vasculogenesis and may even stimulate the immune response. A more recent and novel approach called *adaptive therapy* (Enriquez & Gatenby, 2017; Gatenby, Silva, et al., 2009) advocates administration of cytotoxic drugs at a minimum dose that is necessary to manage symptoms (instead of applying maximum tolerable dose) and adapting the dose depending on how the tumor responds to the therapy. The goal is to replace the "treatment for cure" strategy with a "treatment for stability" approach, where a stable population of chemotherapy‐sensitive cells is maintained, which in turn will suppress the growth of the therapy‐resistant population. This concept borrows heavily from the idea of *combination therapy and evolutionary double bind*, and it is inspired by results of eco‐evolutionary thinking, mathematical modeling and advocates the alternating use of two or more therapeutic agents with the hope that cancer phenotypes resistant to one therapy may still be susceptible to the other therapies (Basanta & Anderson, 2013).

Cancer therapy is an iterative process marred with seemingly unpredictable outcomes, acquisition of imperfect information and layers of uncertainties. How an individual patient will respond to a particular therapy is difficult to forecast and group averages are used as a predictive measure. The underlying explanation for this is principally due to patient heterogeneity, both tumor and nontumor, and our current inability to predict how a drug will be metabolized in a specific patient and how the vast array of tumor cells will respond. When faced with iterative decision making in the face of imperfect information and uncertainties, adaptive management can provide a formal and objective decision‐making process (Nichols et al., 2011; Williams, Nichols, & Conroy, 2002; Williams, Szaro, & Shapiro, 2007). In ecology and resource management, this framework aims to reduce uncertainty by monitoring the state of the system, learning, and adjusting management decisions accordingly. The adaptive management framework can be fruitfully applied to cancer therapy to improve therapeutic outcome and reduce uncertainty by monitoring patients’ response to a therapy, learning, and adjusting therapeutic decisions to achieve better outcomes.

Both the standard of care treatment and the aforementioned alternative approaches focus on targeting and removing or killing cancer cells. However, it is becoming increasingly clear that the tumor microenvironment and ecological interactions between cancer cells, and biotic and abiotic components of the microenvironment play an important role in cancer initiation and neoplastic progression (Ibrahim‐Hashim et al., 2017). The role of microenvironment alteration by cancer via altered energy metabolism in tumorigenesis is well established (Warburg, 1956a). Dynamic reciprocity – the bidirectional interaction between cancer cells and their microenvironment—is believed to initiate cell‐signaling cascades that produce changes in gene expression and cell behavior (Thorne et al., 2015). For instance, cancer‐associated fibroblasts promote tumor growth, invasion, and enhance angiogenesis (Räsänen & Vaheri, 2010; Sun, 2010; Sun, Huang, & Yang, 2015). Valastyan and Weinberg (2011) note that aberrant genetic and epigenetic alterations in tumor cells are insufficient to induce primary tumor progression without microenvironment modifications. Interactions between cancer cells and the metastatic microenvironment are inhibitory during the early stages, but such interactions promote progression toward metastasis in later stages (Klein‐Goldberg et al., 2014). The recognition of the importance of tumor microenvironment, niche construction or modification, and ecological interactions among tumor cells and biotic/abiotic components of the microenvironment has led to the idea of *ecological therapy* (Kareva, 2011a, 2011b; Kareva, Morin, et al., 2015; Pienta, McGregor, Axelrod, & Axelrod, 2008), which advocates targeting not only tumors but also the tumor microenvironment and ecological interactions therein. Finally, *ecological photodynamic therapy* has been suggested to be a novel approach to modulate ecological interactions within tumors aimed at improving therapeutic efficiency (Vittar, Awruch, Azizuddin, & Rivarola, 2010; Vittar, Prucca, Strassert, Awruch, & Rivarola, 2008).

## 6 DEATH BY 1,000 CUTS: A UNIFIED THERAPEUTIC APPROACH TO MANAGING CANCERS

The term “death by a 1,000 cuts” is derived from the Chinese word Lingchi [凌遲], which is translated as a slow process or slow slicing. This was a form of torture and execution that was banned in the early 20th century after being used for nearly 1,000 years. At the heart of Lingchi, and its rendering outside of medieval torture is the notion of imparting several small perturbations, each of which has little effect on its own but collectively demonstrates an additive or synergistic impact. Fundamentally, this is rooted in a central tenet of Integrated Pest Management [IPM], defined as “*…* a decision‐based process involving coordinated use of multiple tactics for optimizing the control of all classes of pests (insects, pathogens, weeds, vertebrates) in an ecologically and economically sound manner” (Prokopy, 2003). The IPM focusses on an adaptive and integrated application of chemical (e.g., pesticides, herbicides), biological (e.g., predators, parasites and other natural enemies), behavioral (e.g., attractants and repellents) and cultural (e.g., crop rotation) approaches to pest control intending to minimize economic loss and the evolution of resistance to pesticides or herbicides (Ehler, 2006; Menalled et al., 2016). Indiscriminate application of chemical control agents, while effective initially, eventually leads to the evolution of resistant genotypes; chemical control of pests or weeds becomes useless at that point. The importance of an eco‐evolutionary and integrated perspective to managing agroecosystems is increasingly being recognized in order to ensure the food security and sustainability of agroecosystems in light of the anthropogenic climate and land‐use changes (Menalled et al., 2016; Thrall et al., 2011). Likewise, it is increasingly recognized that cancer therapies can benefit from ecological‐evolutionary perspectives (Gatenby, Silva, et al., 2009; Maley et al., 2017; Wu, Wang, Ling, & Lu, 2016).

While aggressive radiation or chemotherapy can eradicate a tumor, it may also incapacitate or kill the patient. Sublethal aggressive cytotoxic therapy can select for treatment‐resistant phenotypes that do not respond to the treatment. Given these difficulties, debilitating side effects of cytotoxic therapies and the resilience of tumors, long‐term management of some cancers as a chronic condition using integration of multiple therapeutic approaches may prove to be critical (Kenny & Bissell, 2003). We suggest, just like indiscriminate use of chemical control agents is not effective in controlling pests and weeds in agroecosystems, targeting and killing proliferating cells alone is insufficient to defeat cancer as a disease. Instead, an integrated eco‐evolutionarily sound approach that targets not only the tumor but also the tumor micro and macro‐environment, and interactions between tumor cells and their environments within an adaptive management framework, may produce better outcomes. We propose an ecologically inspired therapeutic approach should seek to:

1. Reduce the evolutionary potential of cancer cells. This can be achieved by adopting strategies that reduce intratumoral diversity, spatial and temporal changes therein, and minimize the potential selection for resistant neoplastic genotypes by maintaining competition between susceptible and resistant genotypes via an adaptive application of cytotoxic agents;
2. Inhibit the proliferative ability of cancer cells. This can be achieved by adopting strategies to discourage niche construction, and depriving neoplasm of resources required for rapid proliferation (e.g., degree of hypoxia, concentration of ATP, glucose and other nutrients, density of blood vessels) (Gupta, Kim, Prasad, & Aggarwal, 2010; Kunnumakkara, Anand, & Aggarwal, 2008; Martuscello et al., 2016; Poff et al., 2017; Woolf et al., 2017);
3. Reduce metastasis by adopting strategies to diminish the survival of circulating cancer cells and their ability to colonize new organs (Langley & Fidler, 2011);
4. Pushing tumors to the edge of chaos thereby creating a state of susceptibility by adopting strategies to disrupt cell‐to‐cell communication and cooperation among cancer cells, accelerating genomic instability or differentiating cells (Hitomi, 2015; Piccirillo et al., 2006);
5. Adopting strategies to minimize the side effects of cytotoxic drugs via adaptive therapies and nutraceuticals (Gaines, Williamson, Hyman, & Kandel, 2017; Goodman & Gardner, 2018; Schwabe & Jobin, 2013); and
6. Adopting approaches that would necessitate tumor cells to make life‐history trade‐offs such that they are forced to choose between proliferation or survival, but not both. For example, rapid proliferation or extended times in sensitive stages of the cell cycle would make them more vulnerable to conventional treatments, whereas enforcing slow‐life history strategies would reduce tumor proliferation rate (Aktipis, 2016; Aktipis, Boddy, Gatenby, Brown, & Maley, 2013; Maley et al., 2017; Nedelcu, 2017).

Ecology‐inspired thinking has led to a more comprehensive understanding of cancer as an eco‐evolutionary process, the recognition of the importance of tumor microenvironments, and the role of complex biotic interactions which could potentially lead to new therapeutic approaches (Gatenby, Brown, et al., 2009; Greaves, 2007; Kareva, Morin, et al., 2015; Kareva, Waxman, et al., 2015; Pienta et al., 2008). Although conventional cancer therapies have been effective in killing cancer cells, this approach has failed to cure cancer because of the evolution of resistance, metastasis, and often debilitating side effects of the cytotoxic therapies. We suggest that eco‐evolutionarily informed therapeutic approaches that combine standard of care treatments with strategies aimed at decreasing the favorability of microenvironment to cancer cell proliferation, and migration and fitness of cancer cells, and reducing the evolution of resistance to cytotoxic therapies may be essential for effectively managing cancer as a chronic condition.

Reductionism and specialization in medical science have contributed to fundamental discoveries on both mechanisms of basic biological systems and in applications of how these systems can be manipulated. However, borrowing from Eastern concepts of *yin and yang*, advancement in one area is often balanced by stagnation in other areas. The blind spot of the reductionist approach is in understanding and managing complex biological systems where multiple interconnected and dependent operations contribute to the fundamental drive of self‐preservation and replication. This is particularly apparent in the area of cancer, which is the poster child for robustness, complexity, and adaptability. While great strides have been made in our knowledge of key contributing factors that initiate and drive cancer progression, compiling this into a comprehensive and efficient management system has challenged us at the clinical level. Cross‐fertilization of scientific disciplines and ideas from one field of science to another has stimulated new paradigms and radical changes that can be viewed as unexplained leaps of logic. However, more often than not, this is more a matter of one's perception or knowledge that is narrowly focused, and incorporating a broader view can result in the “discoveries” of new ideas and approaches that are really “rediscoveries.” The management of complex systems composed of heterogeneous populations of interdependent, interacting and evolving agents has been an area of ongoing study by mathematicians, physicists and ecologists for several decades (e.g., Anand, Gonzalez, Guichard, Kolasa, & Parrott, 2010; Ostfeld, 2011; Sayama, 2015). The similarities between complex ecological systems and a tumor are striking (Table 1). Given the success that ecologists have had in understanding eco‐evolutionary dynamics and managing pests within the IPM framework begs the question: can better outcomes in cancer treatments be achieved if oncologists start to think like ecologists?

## CONFLICT OF INTEREST

None.

## AUTHOR CONTRIBUTIONS

**Brent A. Reynolds:** Conceptualization (equal); investigation (equal); methodology (equal). **Monika W. Oli:** Conceptualization (equal); investigation (equal); methodology (equal); project administration (equal). **Madan K. Oli:** Conceptualization (equal); investigation (equal); methodology (equal).

## Data Availability

There are no data associated with this paper.
